# Vernal keratoconjunctivitis with a limbal mass lesion developing independently of severe papillae formation at the tarsal conjunctiva: a case report

**DOI:** 10.1186/s12886-022-02370-6

**Published:** 2022-03-26

**Authors:** Koichiro Shinji, Taiichiro Chikama, Shun Masuda, Koji Arihiro, Yoshiaki Kiuchi

**Affiliations:** 1 Department of Ophthalmology and Visual Science, Graduate School of Biomedical and Health Sciences, Hiroshima University, 1-2-3 Kasumi, Minami-ku, Hiroshima, 734-8551 Japan; 2 Department of Anatomical Pathology, Hiroshima University Hospital, 1-2-3 Kasumi, Minami-ku, Hiroshima, 734-8551 Japan

**Keywords:** Allergic conjunctivitis, Conjunctival neoplasms, Corneal limbus, Lymphocytes, Immunohistochemistry

## Abstract

**Background:**

A hypertrophic limbal mass lesion is an uncommon finding of vernal keratoconjunctivitis; it normally occurs in eyes with severe papillae formation in the tarsal conjunctiva. We present a case with a limbal mass lesion in a patient with relatively mild allergic findings in the tarsal conjunctiva.

**Case presentation:**

A 12-year-old Japanese boy displaying allergic conjunctivitis presented with a mass lesion at the inferior limbus in the left eye. Relatively mild papillae formation was found on the tarsal conjunctiva in both eyes. We diagnosed that the mass lesion resulted from limbal vernal keratoconjunctivitis and resected it for therapeutic purposes. Histopathological examination showed that eosinophils, lymphocytes, and fibroblasts were present in the subepithelial lesion and the substantia propria of the mass lesion. Immunohistochemical staining detected diffuse and rich infiltration of CD3-positive T lymphocytes and a relatively small number of CD20-positive B lymphocytes and CD138-positive plasma cells that tended to aggregate. The histopathologic features suggested that the limbal mass lesion had similar structures to the papillae at the tarsal conjunctiva of vernal keratoconjunctivitis.

**Conclusion:**

The limbal mass lesion as a finding of vernal keratoconjunctivitis can occur even if the papillae formation at the patient’s tarsal conjunctiva is mild.

## Background

Vernal keratoconjunctivitis (VKC) is a chronic and severe allergic disease involving the tarsal and/or the limbal conjunctiva [1, 2]. Characteristic large cobblestone papillae predominately present at the upper tarsus and are formed by tissue remodeling following prominent inflammatory cellular infiltration [1]. Moreover, the typical findings of limbal VKC are papillae formation and yellow-white points, known as Horner-Trantas’s dots, consisting of degenerating eosinophils and epithelial cell debris [1]. As a relatively uncommon finding, a limbal mass lesion is known to occur in VKC patients [3, 4]. The limbal mass lesion is significantly larger than the typical limbal papillae and consists of plasma cells, histiocytes, eosinophils, and lymphocytes [3, 4]. These components suggest that hypertrophic responses in VKC cause the limbal mass lesion. In the reported cases, the mass lesion occurred at the superior limbus and the affected patients presented large cobblestone papillae at the superior tarsal conjunctiva [3, 4]. Herein, we report a unique case of limbal VKC with a mass lesion occurring at the inferior limbus in a patient with a history of allergic conjunctivitis. Notably, the papillae formation on the patient’s tarsal conjunctiva was relatively mild. To the best of our knowledge, this case report is the first to present immunohistochemical staining of the limbal mass lesion in VKC.

## Case presentation

A 12-year-old Japanese boy with a limbal mass lesion in the left eye was referred to our clinic. The patient had been diagnosed with allergic conjunctivitis and treated with topical administration of pemirolast and/or corticosteroid (fluorometholone or betamethasone) at another clinic for 2 months. According to the referral letter, pruritus, the patient’s first main complaint, had resolved after initiation of topical betamethasone administration, while a limbal mass lesion had appeared during the treatment and developed chronically. The patient had no medical history of atopic disease or previous ocular abnormalities, nor was there a family history of allergies.

**Fig. 1:**
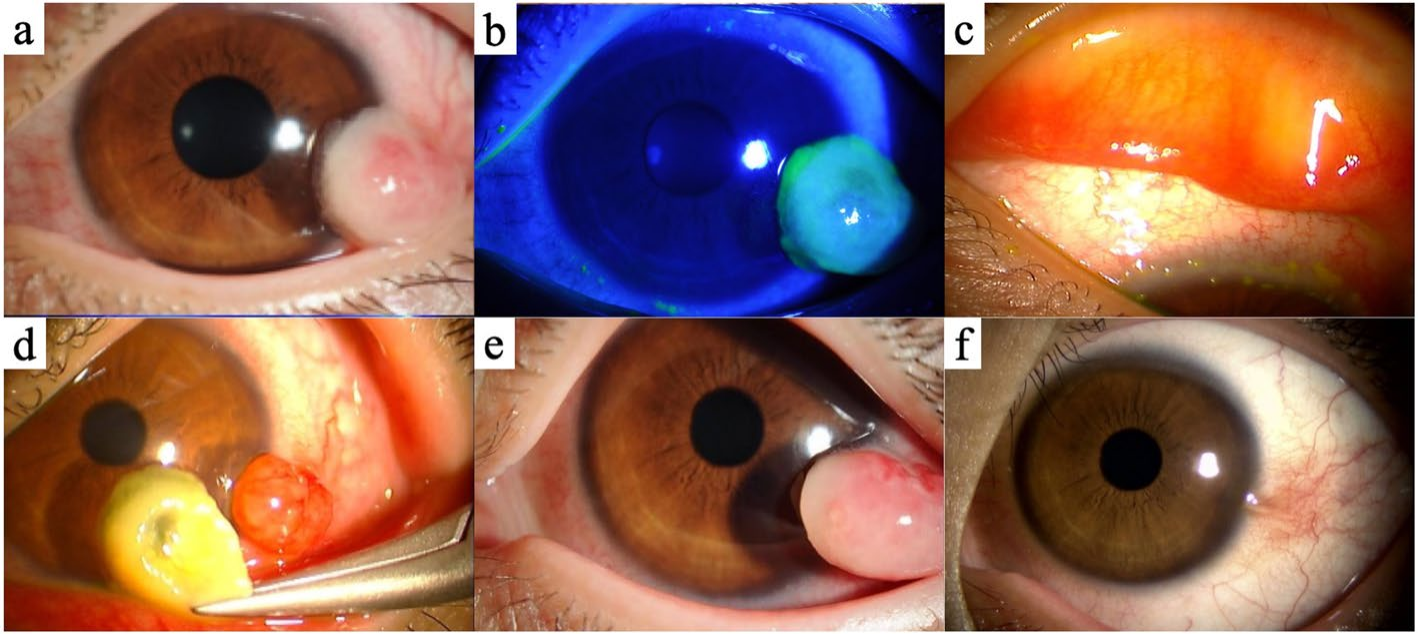
Slit lamp images at the initial and second visits and after the resection surgery. A milky-white colored with partially red appearing mass lesion was observed at the inferior-temporal limbus in the left eye at the initial visit (**a**). No corneal change, including superficial punctate keratitis, was observed in both eyes, even when viewed with a cobalt blue filter (**b**). At the same time, mild papillae formation at the upper tarsal conjunctiva and Horner-Trantas’s dots were observed in the left eye (**c**). The papillae formation was also seen in the right eye. The limbal mass lesion was covered with a pseudomembranous structure, which was readily dissected (**d**). One month after the initial visit, the lesion became larger than at the initial visit (**e**). The limbal mass lesion did not recur over eight years after the resection surgery (**f**)

At the initial visit to our clinic, a noticeable mass lesion, milky-white colored with a partially red appearance, was found at the inferior-temporal limbus in the left eye (Fig. 1a). There was no corneal change including superficial punctate keratitis in both eyes (Fig. 1b). Horner-Trantas’s dots were observed at the superior limbus in the same eye (Fig. 1c). We found mild papillae formation, not as large as cobblestones, at the upper tarsal conjunctiva in both eyes (Fig 1c). The bulbar conjunctiva and the limbus in the right eye were normal. A pseudomembranous structure covered the limbal mass lesion and was readily dissected (Fig. 1d). Given that the patient’s presentation and a medical history that suggested chronic allergic stimuli, we diagnosed that the limbal mass lesion in the left eye was an unusual finding of limbal VKC.

**Fig. 2:**
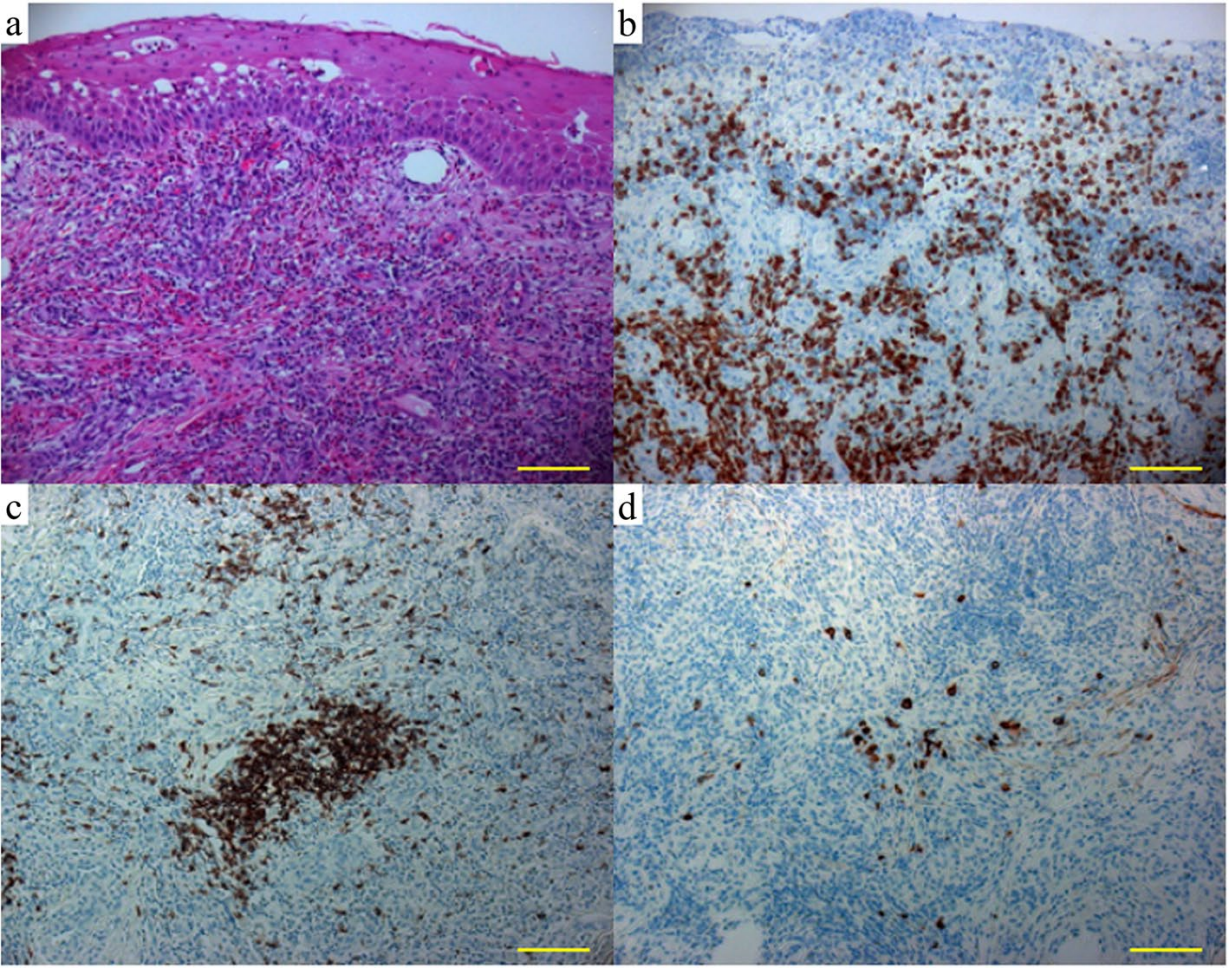
Histopathological and immunohistopathological findings of a limbal mass lesion showed diffuse infiltration of eosinophils and lymphocytes (**a**, hematoxylin-eosin; original magnification ×100). Fibroblasts were also detected. A large number of CD3-positive T lymphocytes were diffusely infiltrated among the subepithelial lesion and the substantia propria (**b**, anti-CD3 immunohistochemistry, 3,3'-diaminobenzidine [DAB] chromogen; original magnification ×100). CD20-positive lymphocytes and CD138-positive plasma cells were present locally and tended to aggregate (**c**, anti-CD20 immunohistochemistry, DAB chromogen; original magnification ×100. **d**, anti-CD138 immunohistochemistry, DAB chromogen; original magnification ×100.). All images were taken with the equipment as follows; microscope: Axioskop 2 Plus (Carl Zeiss, Oberkochen, Germany), objective lens: Plan-Neofluar (Carl Zeiss), and camera: Axiocam (Carl Zeiss). Scale bar: 100 μm

Because the patient did not complain of any subjective symptoms such as pruritus, we stopped the topical corticosteroid administration and continued only with that of pemirolast as a mast cell stabilizer after dissection of the pseudomembranous structure. However, one month later, the mass lesion became larger than that at the initial visit, measuring 4 × 4 × 4 mm (Fig. 1e). We resected the limbal mass lesion for therapeutic purposes and sent the obtained specimen for pathological analysis. Hematoxylin-eosin staining detected rich infiltration of eosinophils and lymphocytes in the subepithelial lesion and the substantia propria of the mass lesion (Fig. 2a). Fibroblasts were also observed in both areas (Fig. 2a). Immunohistochemical staining detected that CD3-positive T lymphocytes had predominantly and diffusely infiltrated in the subepithelial lesion and the substantia propria of the mass lesion (Fig. 2b). By contrast, relatively small numbers of CD20-positive B lymphocytes and CD138-positive plasma cells were locally detected and tended to aggregate (Fig. 2c and d). These histopathological findings supported our diagnosis that the limbal mass lesion was a hypertrophic response induced by chronic allergic stimuli, rather than by ocular squamous neoplasia [5].

The patient’s postoperative course was uneventful. We have been using topical administration of corticosteroids (betamethasone or fluorometholone) and/or that of tacrolimus to control the allergic conjunctivitis. The limbal mass lesion did not recur over 8 years (Fig. 1f).

## Discussion and conclusions

To the best of our knowledge, only two cases of a limbal mass lesion occurring in VKC patients have been reported. The lesion’s morphology of one case mimicked ocular surface squamous neoplasia [3], while the other appeared similar to our case [4]. In both previous cases, typical cobblestone papillae were observed on the upper tarsus and the mass lesion was present at the superior limbus. These findings led to a hypothesis that rubbing by the large cobblestone papillae at the upper tarsal conjunctiva might mechanically aggravate inflammation of the bulbar conjunctiva and result in the limbal mass formation [3]. However, this hypothesis may not be true for our case; because papillae formation on the upper tarsal conjunctiva was relatively mild and the mass lesion was located at the inferior limbus. The clinical presentation of our case may indicate that the limbal mass lesion occurred without rubbing by the cobblestone papillae at the tarsal conjunctiva.

The differential diagnoses we supposed were dermoid, conjunctival papilloma, or ocular squamous neoplasia. However, dermoid, a congenital tumor [6], was excluded because no conjunctival tumors were previously evident. Moreover, the patient in our case was too young for an age-related predisposition for conjunctival papilloma [7]. Although ocular squamous neoplasia could not be absolutely excluded at the time, the pseudomembranous structure was unlikely to have occurred as a result of such a non-inflammatory disease.

Histopathological studies showed that the limbal mass lesion of our case consisted of eosinophils, lymphocytes, plasma cells, and fibroblasts, corresponding to the previous reports [3, 4]. It must be noted that a considerable number of T lymphocytes infiltrated among the subepithelial lesion and the substantia propria of the mass lesion in our case. An increase of T lymphocytes is a common feature in the conjunctiva of VKC patients [1]. Furthermore, locally aggregated B lymphocytes were detected from our specimen. B lymphocyte aggregation was also observed in papillae at the tarsal conjunctiva in VKC patients [8, 9]. Therefore, the histopathologic features exhibited in our case may indicate that the mass lesion had a similar pathogenesis to the papillae formation at the tarsal conjunctiva in VKC patients.

As a limitation, we acknowledge that this report describes only one case, and thus it cannot be generalized that the severe papillae formation at the tarsal conjunctiva does not affect the limbal mass formation in VKC patients. Further studies are required to investigate the interaction between the tarsal and the limbal conjunctiva in affected patients.

In conclusion, we found a mass lesion at the inferior limbus in a patient with allergic conjunctivitis with relatively mild papillae formation at the tarsal conjunctiva. Histopathological features of the mass lesion were consistent with VKC. In the clinical examination of a patient with a limbal mass lesion, the possibility of limbal VKC should be considered, even if the patient does not display the typical tarsal changes observed in VKC.

## Data Availability

All the data generated or analyzed during this study are included in this published article.

## Abbreviation

VKC: Vernal keratoconjunctivitis.
